# A Deleterious Mutation in *DNAJC6* Encoding the Neuronal-Specific Clathrin-Uncoating Co-Chaperone Auxilin, Is Associated with Juvenile Parkinsonism

**DOI:** 10.1371/journal.pone.0036458

**Published:** 2012-05-01

**Authors:** Simon Edvardson, Yuval Cinnamon, Asaf Ta-Shma, Avraham Shaag, Yang-In Yim, Shamir Zenvirt, Chaim Jalas, Suzanne Lesage, Alexis Brice, Albert Taraboulos, Klaus H. Kaestner, Lois E. Greene, Orly Elpeleg

**Affiliations:** 1 Monique and Jacques Roboh Department of Genetic Research, Hadassah, Hebrew University Medical Center, Jerusalem, Israel; 2 Laboratory of Cell Biology, National Heart, Lung, and Blood Institute, National Institutes of Health, Bethesda, Maryland, United States of America; 3 Bonei Olam, Center for Rare Jewish Genetic Disorders, Brooklyn, New York, United States of America; 4 CRICM, University Pierre et Marie Curie, INSERM, UMR_S975, CNRS UMR 7225, Hospital Pitié-Salpêtrière, Paris, France; 5 IMRIC, The Hebrew University-Hadassah Medical School, Jerusalem, Israel; 6 Department of Genetics, Institute for Diabetes, Obesity and Metabolism, University of Pennsylvania School of Medicine, Philadelphia, Pennsylvania, United States of America; Centre Hospitalier Universitaire Vaudois (CHUV), Switzerland

## Abstract

Parkinson disease is caused by neuronal loss in the substantia nigra which manifests by abnormality of movement, muscle tone, and postural stability. Several genes have been implicated in the pathogenesis of Parkinson disease, but the underlying molecular basis is still unknown for ∼70% of the patients. Using homozygosity mapping and whole exome sequencing we identified a deleterious mutation in *DNAJC6* in two patients with juvenile Parkinsonism. The mutation was associated with abnormal transcripts and marked reduced DNAJC6 mRNA level. *DNAJC6* encodes the HSP40 Auxilin, a protein which is selectively expressed in neurons and confers specificity to the ATPase activity of its partner Hcs70 in clathrin uncoating. In Auxilin null mice it was previously shown that the abnormally increased retention of assembled clathrin on vesicles and in empty cages leads to impaired synaptic vesicle recycling and perturbed clathrin mediated endocytosis. Endocytosis function, studied by transferring uptake, was normal in fibroblasts from our patients, likely because of the presence of another J-domain containing partner which co-chaperones Hsc70-mediated uncoating activity in non-neuronal cells. The present report underscores the importance of the endocytic/lysosomal pathway in the pathogenesis of Parkinson disease and other forms of Parkinsonism.

## Introduction

Parkinson's disease (PD) is an insidious and progressive neurodegenerative disorder causing slowed movement, tremor, rigidity and postural instability. The disease is characterized by neuronal loss in the substantia nigra and other brain regions, and is usually associated with the formation of intracellular protein inclusions in damaged neurons, known as Lewy bodies. Several genes known to function in the endocytic/lysosomal pathway or in mitochondrial repair/elimination machinery have been implicated in the pathogenesis of PD. At present, known Mendelian forms and genetic risk factors of PD explain only about 30% of the disease risk at the general population level [1]. While familial forms of PD and Juvenile variants are rare, the identification of their disease-causing genes is important as they highlight specific pathways and because common genetic variants in these genes may confer a risk of developing the sporadic disease. Here, we report a homozygous mutation in *DNAJC6* in two patients with autosomal-recessive juvenile Parkinsonism.

## Results

**Figure 1:**
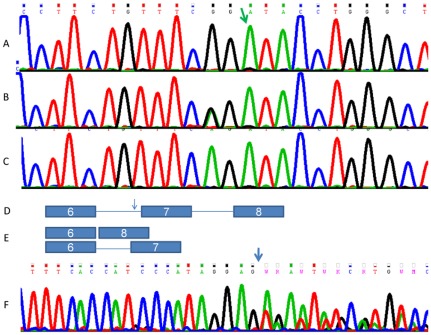
The c.801 −2 A−>G mutation in the DNAJC6 gene. The green arrow points at the first nucleotide of exon 7 and the mutation affects the preceding AG splice acceptor site of intron 6 which is changed to GG in the patient (A). The sequence of an obligate heterozygote is shown in (B) and that of a control in (C). Schematic representation of the mutation site at the genomic level (D) and its impact on the cDNA (E). Chromatogram of cDNA from a patient encompassing the 3′ junction of exon 6 (F) and demonstrating a transcript lacking exon 7 and another transcript where nNext to the last base of exon 6 (blue arrow) overlapping exon 8 sequence is the intronic sequences from intron 6 (c.801 −91). The normal exon 6/exon 7 spliced form is undetectable.

In order to localize the mutated gene in this family we searched for homozygous regions common to the two patients but not to their healthy brother, by genotyping dense DNA SNP arrays. This analysis resulted in identification of eight homozygous genomic regions of more than 2 Mb each, totaling 102.75 Mb. These regions encompass about 800 protein-coding genes, making the identification of plausible candidate genes difficult. We therefore performed whole exome sequencing of patient II-2 sample. This analysis resulted in the identification of 18,494 coding variants (single-nucleotide variants and small insertions and deletions) of which 7,387 variants were homozygous, but only 740 homozygous coding or splice site variants were present in the eight homozygous regions. Thirty variants were not annotated in dbSNP132, in the 1,000-genome or in our in-house database, and 15 remained after filtering out synonymous changes. Sanger sequencing confirmed only 11 changes and these segregated with the disease within the family. However, out of the 11 variants, ten were annotated in dbSNP135. We further checked for their conservation score GERP (obtained via SeattleSeq Annotation website). The score of six variants was above 3.0 and these were tested for their potential pathogenicity using Polyphen, SIFT, and Mutation taster software. Three variants were reported by these tools as potentially pathogenic: Arg141Cys mutation in *POLR1C* (rs148385032), Cys3346Arg in *PKHD1* (rs149798764), and c.801 −2 A>G mutation in *DNAJC6* (at chr.1:65623981). Mutations in *POLR1C* were recently shown to cause Treacher Collins syndrome [2] and *PKHD1* mutations are associated with polycystic kidney and hepatic disease [3] and were thus excluded as candidate genes for PD. Of note the index case had normal kidneys as per abdominal ultrasound and did not display the facial characteristics of Treacher Collins syndrome. The c.801-2 A−>G mutation in the *DNAJC6* gene segregated with the disease state within the family; both patients were homozygous, while the parents and two healthy siblings were heterozygous for the mutation; one sister was homozygous for the normal allele (figure 1A–C). The mutation was not carried by any of 208 anonymous ethnic matched controls, neither was it present in the data of the 5379 Exomes available at the NHLBI Exome Sequencing Project website Release Version: v.0.0.9.

**Figure 2:**
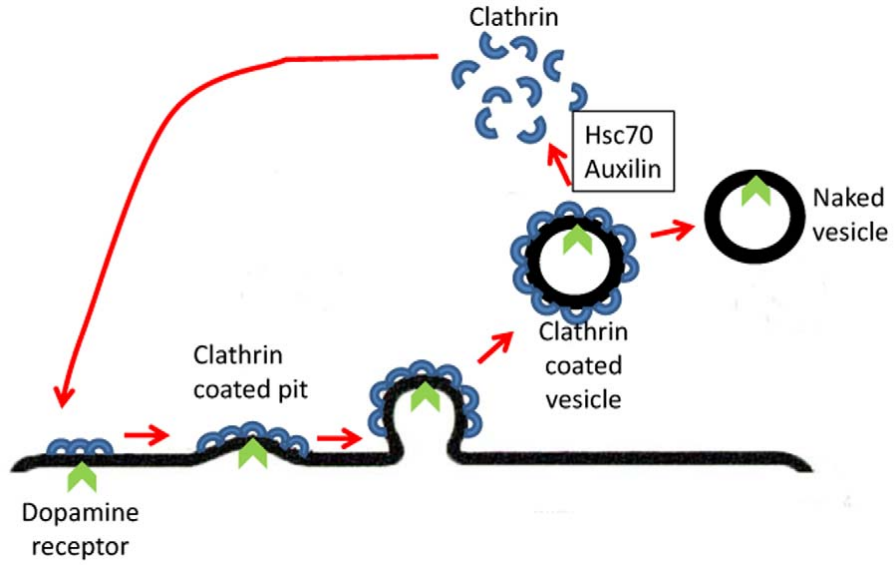
Schematic representation of clathrin-mediated endocytosis. Plasma membrane molecules (in this case the dopamine receptor) associate with nascent clathrin-coated pits which then mature invaginate and finally pinch off to form clathrin-coated vesicles. The shedding of the coat takes place after the vesicle buds from the plasma membrane. This process is driven by Hsc70 ATP hydrolysis activity which is recruited to clathrin coats by Auxilin. The uncoated vesicle fuses with the membrane of a target compartment and delivers its cargo. Clathrin molecules are directed to the plasma membrane for re-use.

*DNAJC6* encodes Auxilin which belongs to the evolutionarily conserved DNAJ/HSP40 family of proteins [4]. These proteins regulate molecular chaperone function by stimulating ATPase activity in many cellular processes and Auxilin functions specifically in the Clathrin Mediated Endocytosis (CME) pathway (figure 2). *DNAJC6* consists of 19 exons which encode 970 amino acids. The effect of the mutation on cDNA was studied in RNA from the lymphoblast cell line and from cultured skin fibroblasts of patient II-4. Homozygosity for the c.801 −2 A−>G mutation resulted in the generation of two mis-spliced cDNA transcripts; an in-frame exon 7-skipped transcript lacking amino acids 268–328, and an out-of-frame transcript with an insertion of the last 91 nucleotides of IVS 6 (c.801 −91) between exon 6 and 7, resulting in the addition of eight non-synonymous residues before reaching a termination codon. Importantly, the normally-spliced transcript was undetectable (figure 1E–F). This is suggestive but does not confirm pathogenicity of the c.801 −2 A−>G mutation within the family. Nonetheless, the ratio of *DNAJC6* cDNA normalized to beta-actin cDNA of five controls was 0.064+/−0.007 whereas the ratio was 0.011 in the patient, indicating a significant instability of the DNAJC6 mRNA in the patient's cells.

## Discussion

CME is a major pathway for the internalization of selected protein cargo from the plasma membrane to membrane-bound internal compartments. The cargo, mainly receptors and their bound ligands, is recruited to nascent clathrin-coated pits, which then mature, invaginate, and ultimately undergo fission to produce Clathrin-Coated Vesicles (CCVs) [5], [6]. Once the vesicle has pinched off from the plasma membrane, the coat is lost and its components are recycled. The vesicle itself then fuses with the membrane of a target compartment. The shedding of the coat, which occurs almost immediately after the endocytic CCV buds from the plasma membrane, is driven by ATP hydrolysis and is important for clathrin recycling [7], [8]. The necessary ATPase activity is contributed by Hsc70, which like all Hsp70 homologues, interacts with J-domain containing cochaperones that specify its targets. In the case of clathrin coats, the relevant J-domain protein is Auxilin [9]. Three functional domains are instrumental for Auxilin's co-chaperone activity: an N-terminal PTEN-like domain (residues 40–421), which is important for recruitment of Auxilin onto CCVs, a middle domain which binds clathrin, and a carboxyl-terminal J-domain (residues 886–931) which binds Hsc70 [10], [11]. The low mRNA level in our patients and the production of two abnormal transcripts lacking either a significant part of the PTEN-like domain or the carboxyl-terminal J-domain, suggest that cells homozygous for the c.801-2 A−>G mutation lack the Auxilin protein.

Auxilin is selectively expressed in neurons and is enriched in nerve terminals [12]. In non-neuronal cells, another J-domain containing partner, the ubiquitously expressed cyclin-G-dependent kinase (GAK), co-chaperones Hsc70-mediated uncoating activity on CCV [13]. Reduction of Auxilin and its homologue GAK was shown to result in the impairment of CME and of the clathrin-dependent traffic of cargo from the Golgi to the lysosome [14]. Nonetheless, Auxilin is not redundant in mice and Auxilin null mice exhibit an abnormally increased retention of assembled clathrin on vesicles and in empty cages despite upregulation of GAK. This defect, in turn, leads to impaired synaptic vesicle recycling and perturbed CME, probably because of sequestration of clathrin coat components and their accessory factors with failure to form new clathrin-coated pits [15]. Mice lacking Auxilin have a high rate of unexplained early postnatal mortality, and surviving pups fail to thrive though they do have normal life span. We examined brain tissue of these Auxilin null mice but could not detect any alteration in substantia nigra morphology, dopamine transporter abundance or distribution (data not shown), in agreement with the lack of any gait or movement abnormalities in the mutant mice. Lack of neurodegeneration in transgenic mice that express mutated versions of PD genes is a recognized phenomenon in PD research [16].

The identification of the molecular basis of familial PD and juvenile variants is still in progress [17]. Loss of-function mutations in *PRKN, PINK1*, and *DJ-1*, all implicated in mitochondrial repair/elimination machinery, give rise to a pure, early onset PD. Another group of PD-causing genes participate in the endosomal/lysosomal pathway. α-synuclein, encoded by *SNCA*, was shown to participate in synaptic vesicle formation and recycling and to facilitate CME [18]. The *C. elegance* orthologue of LRRK2, another central player in PD pathogenesis, regulates the proper localization of synaptic vesicles in neurons [19]. Dominant mutations in PD patients were recently identified in *VPS35*, a component of the retromer complex which mediates retrograde transport between endosomes and the trans-Golgi network [20], [21]. Another player in the endosomal/lysosomal compartment, ATP13A2, is also associated with PD [22]. Finally, mutations in the gene encoding the lysosomal enzyme glucocerebrosidase, which interacts with α-synuclein [23], have also been identified as PD susceptibility alleles [24]. The involvement of these five genes underscores the role of the endosomal/lysosomal pathway in PD pathogenesis. We propose that Auxilin is a new endosomal/lysosomal Parkinsonism-related gene. We refrain from the term “Parkinson Disease" since the patients did not respond to L-Dopa. Since dopamine receptors undergo CME [25] followed by endosomal sorting to recycling or degradation [26], it is conceivable that a homozygous deleterious mutation in the Auxilin encoding gene, *DNAJC6*, would give rise to abnormal dopamine receptor metabolism with the resultant parkinsonism. We studied the endosomal system in fibroblasts of patient II-2 by measuring transferrin uptake but observed no differences in either the levels or the pattern of transferrin uptake between control and mutant cells suggesting that in human fibroblasts Auxilin is redundant and its activity overlaps that of GAK.

*DNAJC6* does not fall within any of the peaks discovered by recent PD genome-wide association studies. Focused studies on common variation in this gene are perhaps now warranted [27]. We determined the sequence of the coding exons and splice sites of *DNAJC6* in 15 patients with PD onset before 40 years of age but could not find any pathogenic mutation.

In summary, using homozygosity mapping in a consanguineous small family, followed by whole exome sequencing of a single patient who suffered from juvenile Parkinsonism, we were able to identify a new Parkisnsonism-related gene. It is likely that *DNAJC6* is indispensible in the human substatia nigra but is redundant in peripheral tissues. Nevertheless, the findings presented above are important for the genetic counseling of the patient's extended family, and emphasize the role of CME in the pathogenesis of PD.

## Materials and Methods

### Clinical description

**Figure 3:**
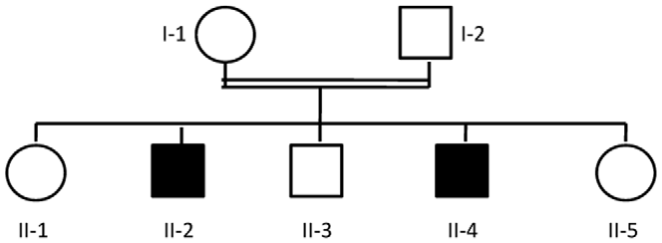
Family pedigree. The patients are represented by filled symbols.

The proband, patient II-4, presented at 13 years of age along with his 18 year old brother, patient II-2.The two brothers were the sons of first cousin Arab-Moslem parents of Palestinian origin (figure 3). The parents and three siblings were healthy. Pregnancy, delivery and early psychomotor development of the two patients was normal. On physical examination at 11 years, patient II-4 was first noted to have debilitating bradykinesia, rigidity, postural instability, hypomimia, and asymmetric tremor at rest. Therapeutic trials with Amantadine, Pramipexole, and L-Dopa did not provide any relief and the patient became wheel-chair bound at age 13. Despite being cognitively normal he could not attend school due to his physical impairment. Patient II-2 was reported to suffer from identical symptoms during childhood and had deteriorated to a dependent state by age 18. The course of his disease was more insidious with bradykinesia noted at 7 years and later appearance of rigidity, tremor and postural instability. The physical examination of both patients revealed hypomimia, slow and dysarthric speech, bradykinesis, pill-rolling tremor at rest, and postural instability with inability to walk. Glabellar tap was unextinguishable and no gaze paresis was elicited. In patient II-2 hypometric saccades were noted. Tone was increased in limbs with no spasticity, pyramidal signs or dystonia. Deep tendon reflexes were symmetric and normal. Plantar reflexes were downgoing. Fine alternating movements, finger and foot tapping were slow and reduced in amplitude. No cerebellar or sensory deficits were found. Brain Magnetic Resonance Imaging (MRI) was unremarkable in both patients. The parents, another brother and two sisters were healthy at ages 11 to 21 and had normal neurologic examinations.

The study was approved by the Hadassah Institutional Review Board and the Ministry of Health. The parents consented to participate.

### Genetic mapping

Single nucleotide polymorphism (SNP) genotyping was performed in the DNA samples of the two patients and an unaffected brother, with the Affymetrix GeneChip Human Mapping 250 K Nsp Array as previously described [28]. Homozygous regions larger than 2 Mb were manually searched. Selected SNP markers were used for genotyping the remaining family members. The carrier rate of the pathogenic mutation was determined by Sanger sequencing of the relevant exon in DNA samples of 133 anonymous ethnic matched controls.

### Exome sequencing

The DNA sample of patient II-2 was enriched for exonic sequences with the SureSelect Human All Exon v.2 Kit, which targets 44 Mb (Agilent, Santa Clara, CA, USA). Sequencing was carried out on a GAIIx (Illumina, San Diego, CA, USA) with 100-bp paired-end runs. Image analysis and base calling were performed with the Genome Analyzer Pipeline version 1.5 using default parameters. The sequence reads were aligned to human genome assembly hg18 (GRCh36) with DNAnexus software (Palo Alto, CA) using default parameters.

### mRNA quantification

Total RNA was isolated from fibroblasts of patient II-4 and from five normal unrelated adult controls, using Tri Reagent (Sigma). DNA traces were removed by treatment with TURBO DNAse kit (Ambion). RNA was reversed transcribed using Improm II kit (Ambion) and random hexamers primers. DNA concentration of two plasmids, one with an ACTB insert and the other with a cDNA insert encompassing exon 4 and 5 of DNAJC6, was determined by spectrophotometer. Serial dilution of the plasmids' DNA was performed in order to create a calibration curve on real time PCR instrument (ABI 7900). These calibration curves were used to determine the copy number of the respective transcripts in RNA samples from the patient and the controls. We used the concentration of the ACTB cDNA to normalize the concentration of the DNAJC6 cDNA and each sample was PCR four times.

### Transferrin uptake in fibroblasts

In order to study the endosomal system, transferrin uptake was measured in fibroblasts of patient II-2 as previously described [14] with some modifications. Cells were incubated for 1 h at 37°C in labeling medium (F12 containing 10 mM HEPES, pH 7.3 and 0.2% w/v BSA) to remove unlabeled transferrin. They were then labeled for 1 h on ice with 50 µg/ml Alexa fluor 488-transferrin (Invitrogen) in labeling medium. After rinsing twice with warm labeling medium, they were incubated at 37°C for either 20 or 40 min to allow transferrin uptake. The cells were then fixed with 2% formaldehyde in PBS (RT, 30 min) and examined with a Zeiss Axiovert 200 microscope equipped with a 100× oil immersion objective.

### Web resources

Online Mendelian Inheritance in Man (OMIM) http://www.omim.org/ SeattleSeq Annotation website - http://snp.gs.washington.edu/SeattleSeqAnnotation/ PolyPhen-2 prediction of functional effects of human nsSNPs http://coot.embl.de/PolyPhen/ SIFT - Sorting Tolerant From Intolerant http://sift.jcvi.org/ Mutation taster - http://www.mutationtaster.org/ NHLBI Exome Sequencing Project Exome Variant Server - http://evs.gs.washington.edu/EVS/.
